# Supplementary material for: Electroassisted Incorporation of Ferrocene Within Sol–Gel Silica Films to Enhance Electron Transfer—Part II: Boosting Protein Sensing with Polyelectrolyte-Modified Silica
Source: Molecules. 2025 Aug 2;30(15):3246. doi: 10.3390/molecules30153246 (PMC12348292; doi:10.3390/molecules30153246)
Supplement: Supplementary file 1 [file molecules-30-03246-s001.zip › molecules-3754971-supplementary.pdf]

Supporting information for:

Electroassisted Incorporation of Ferrocene within Sol–Gel Silica Films to Enhance Electron Transfer. Part II: Boosting Protein Sensing with Polyelectrolyte-Modified Silica

Rayane-Ichrak Loughlani [a,b], Alonso Gamero-Quijano [a,c] and Francisco Montilla [a]

Fig. S1 presents a schematic illustration of the electroassisted accumulation process of ferrocene species within the sol-gel hybrid silica films. The ITO/silica-PSS and ITO/silica-PDADMAC electrodes were immersed in a 0.3 mM $FcPF_6$ Trizma aqueous solution, and multiple potential cycles were applied to drive the electroassisted incorporation of ferrocene into the silica matrix. During this process, ferrocene species were progressively confined within the charged, porous network of the hybrid film. After stabilization of the cyclic voltammograms, the electrodes were removed from the solution and referred to as Fc@hybrid silica-modified electrodes.

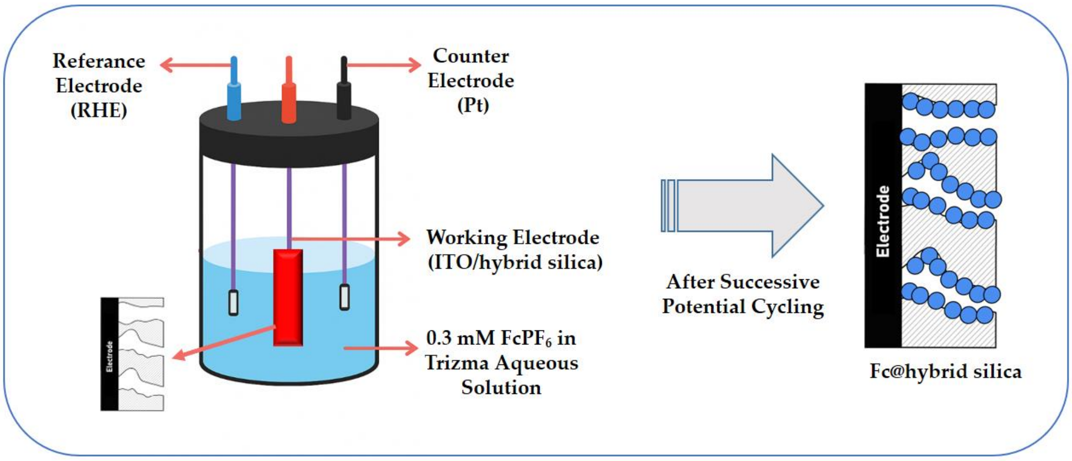

Figure S1. Schematic representation of the electroassisted accumulation process of ferrocene species within the hybrid silica film.

Fig. S2 shows the N_2 adsorption isotherms and the pore width distribution for a silica-PSS and silica-PDADMAC samples.

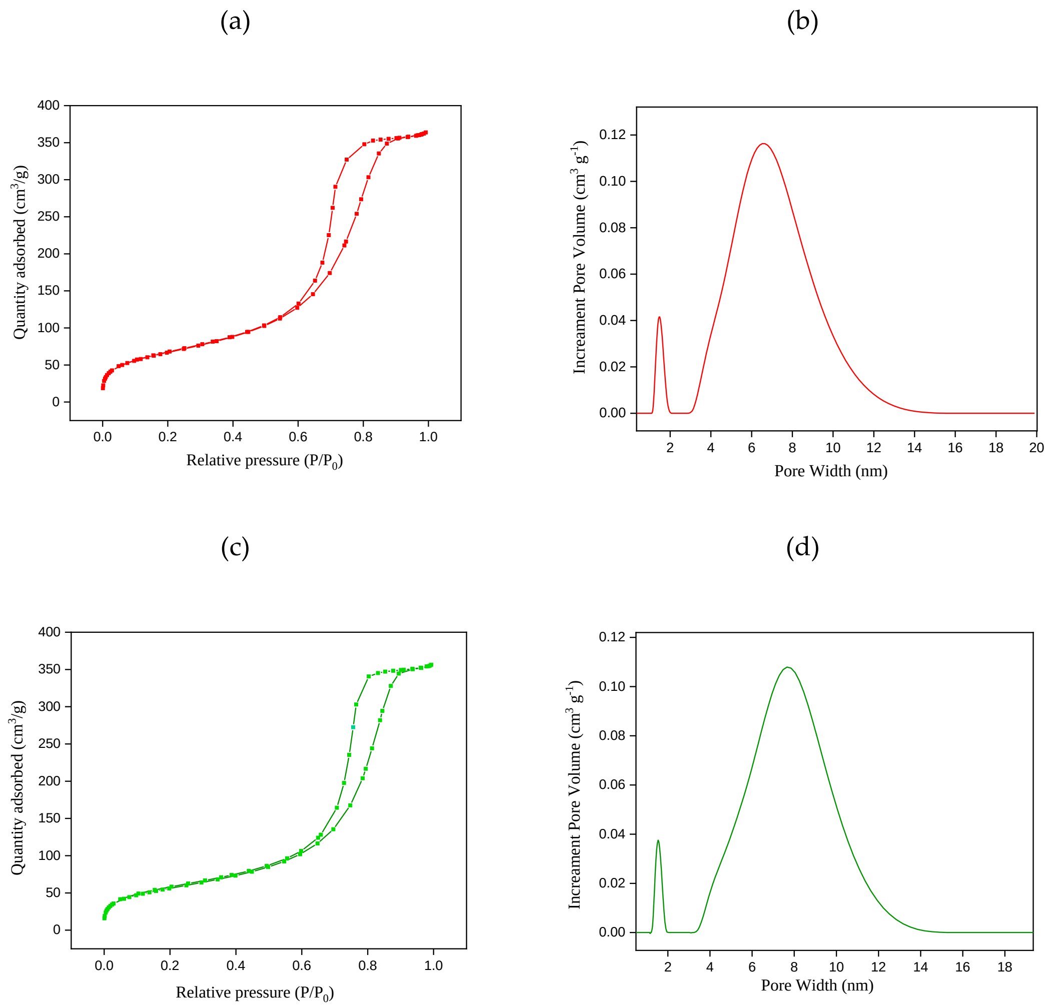

Figure S2: (a, c) are respectively the N_2 adsorption isotherms at 77.35 K for a silica-PSS and silica-PDADMAC sample. (b, d) are respectively the pore width distributions for a silica-PSS and silica-PDADMAC sample.

According to the IUPAC classification, the silica-PSS and silica-PDADMAC samples present type IV adsorption isotherms[36,37]. The microporous volumes obtained from N_2 adsorption indicate that the material has a micropore size.

The plot of pore width distribution for silica-PSS sample indicates the presence of micropores with a pore size of 1.46 nm and mesopores with a pore size of 6.5 nm. The plot of pore width distribution for the silica-PDADMAC sample indicates the presence of micropores with a pore size of 1.56 nm and mesopores with a pore size of 7.7 nm.

The particulate properties of the silica-PSS and silica-PDADMAC samples, such as surface area, pore size, and volume distribution, are listed in the table S1.

Table S1. Textural properties of the silica-PSS and silica-PDADMAC samples obtained from the N_2 adsorption isotherms.

Sample	S_{BET} (m^2 g^{-1})	V N_2 (cm^3 g^{-1})	Ads P_D (nm)	Des P_D (nm)
Silica-PSS	243.49	0.5	5.00	6.50
Silica-PDADMAC	204.17	0.55	5.99	7.62

Fig. S3 shows the stabilized cyclic voltammograms recorded for the ITO/silica-PSS and ITO/silica-PDADMAC modified electrodes in an aqueous solution containing 0.3 mM ferrocenium hexafluorophosphate ($FcPF_6$) in Trizma buffer, at various scan rates ranging from 10 to 1000 mV s^{-1}.

At a scan rate of 10 mV s^{-1}, the voltammogram recorded with ITO/silica-PSS (See Fig. S3a) displays a single anodic peak with a bell-shaped profile centered at 1.23 V, corresponding to the oxidation of neutral ferrocene to ferrocenium. The reverse process appears as a cathodic peak centered at 0.78 V, yielding a peak-to-peak separation of approximately 500 mV. This wide separation suggests sluggish electron transfer kinetics and transport limitations within the silica-PSS film.

As the scan rate increases to 20 mV s^{-1}, the peak-to-peak separation increases by approximately 30 mV. At a scan rate of 50 mV s^{-1}, a shoulder or pre-peak emerges around 0.98 V on the anodic wave. This newly observed feature is associated with the oxidation of ferrocene species located in the inner layers of the silica matrix. Changes in the shape of the anodic peak become more pronounced at higher scan rates (500 and 1000 mV s^{-1}), indicating increasing kinetic and transport limitations within the silica-PSS film (See Fig. S3b).

A similar electrochemical response is observed for the ITO/silica-PDADMAC electrode (Figs. S3c and S3d). At low scan rates (10-20 mV s^{-1}) well-defined redox peaks are observed. However, at higher scan rates, the voltammograms exhibit broader peaks and increased peak-to-peak separation, along with features indicative of ferrocene oxidation occurring at different depths within the matrix, such as the appearance of a pre-peak around 1.0 V at a scan rate of 50 mV s^{-1}. These observations confirm that, as with the silica-PSS film, Fc species confined within the PDADMAC-containing film also experience mass transport limitations and sluggish electron transfer at elevated scan rates.

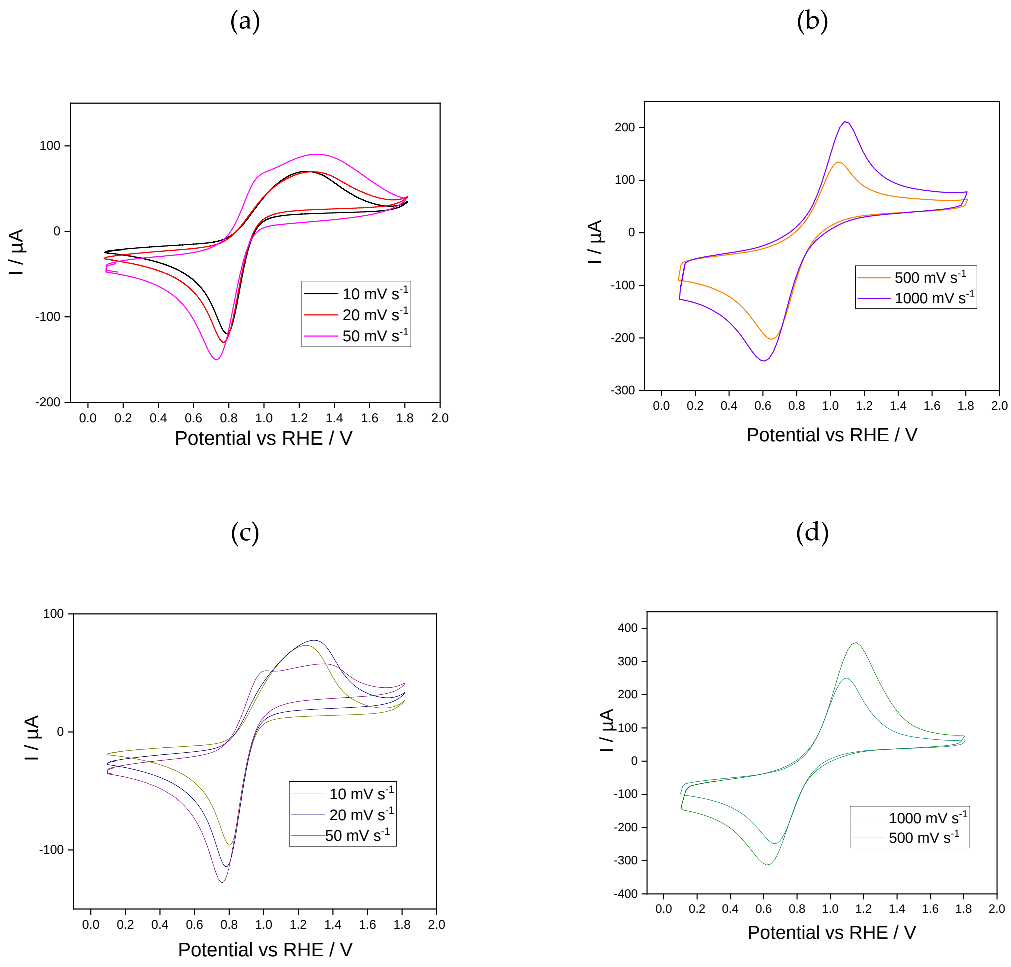

Figure S3. Stabilized cyclic voltammograms recorded for (a, b) an ITO electrode modified with silica-PSS and (c, d) an ITO electrode modified with silica-PDADMAC in an aqueous solution containing 0.3 mM ferrocenium hexafluorophosphate ($FcPF_6$) + Trizma buffer (pH 8.44). Measurements were performed at various scan rates. The thickness of the deposited films was approximately 200 µm.

Fig. S4 shows a schematic illustration of the rinsing process with ultrapure water following the electroassisted accumulation of ferrocene species (as depicted in Figure S1). This step is intended to remove loosely retained or unbound ferrocenium species from the hybrid silica film, ensuring that only deeply confined species remain within the matrix prior to further electrochemical characterization.

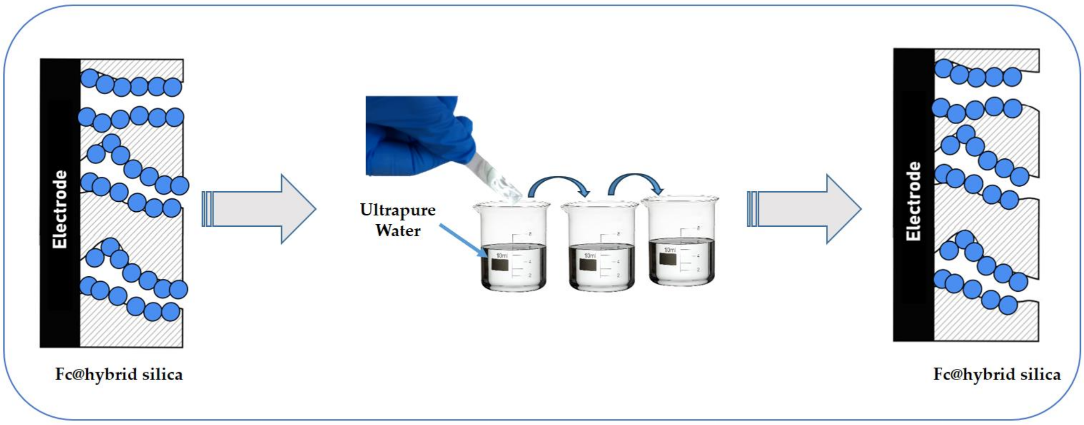

Figure S4. Schematic illustration of the rinsing process with ultrapure water to remove loosely retained ferrocenium species from the hybrid silica film after electroassisted accumulation, represented in Figure S1.

Fig. S5 shows a schematic representation of the electroassisted desorption process of ferrocene species from the hybrid silica film. After rinsing (presented in Figure S4), the Fc@hybrid silica-modified electrode was immersed in a ferrocenium-free Trizma buffer solution, and cyclic voltammetry was applied. During repeated potential cycling, a gradual release of ferrocene species from the film was induced. Once a stabilized voltammogram was obtained, the electrode was removed from the electrochemical cell.

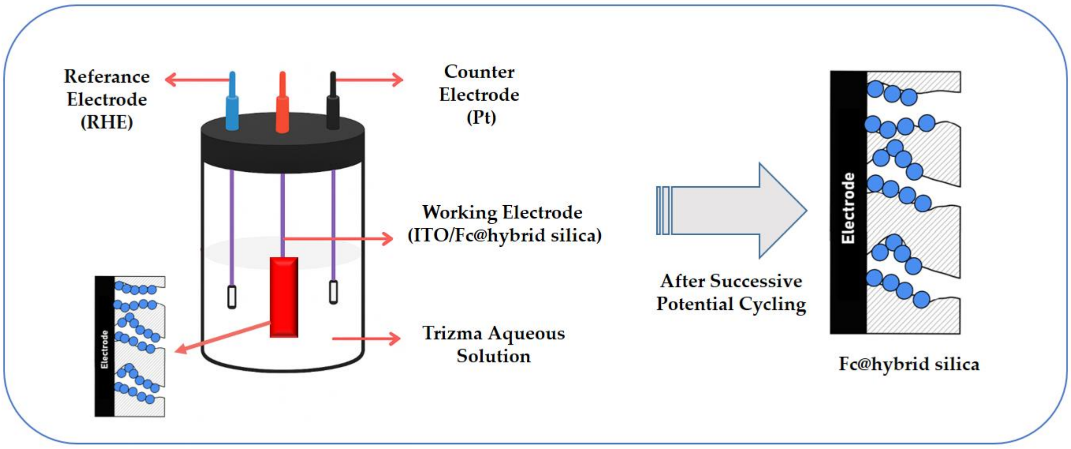

Figure S5. Schematic representation of the electroassisted desorption of ferrocenium species from the hybrid silica film in ferrocenium-free solution.

Fig. S6 shows the stabilized cyclic voltammograms recorded for bare ITO, ITO/silica-PSS, and ITO/silica-PDADMAC electrodes in a 0.1 M Trizma buffer solution (pH 8.44). All three electrodes exhibit a featureless double-layer charging current, with no observable faradaic peaks within the studied potential window, indicating the electrochemical stability of the films in the absence of redox-active species. The close similarity in current profiles between the modified and unmodified electrodes suggests that the deposition of the silica-polyelectrolyte films does not significantly affect the interfacial resistance or charge transfer characteristics of the underlying ITO substrate.

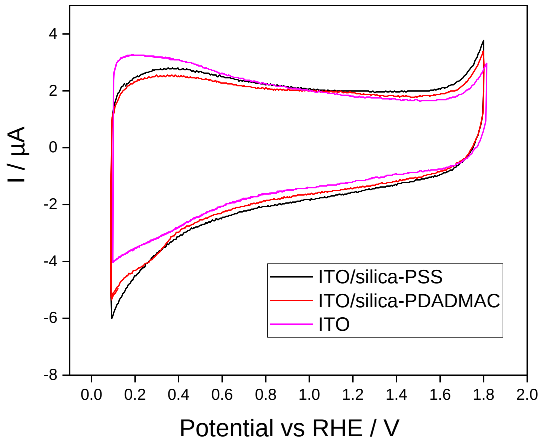

Figure S6. Stabilized cyclic voltammograms recorded for bare ITO, ITO/silica-PSS, and ITO/silica-PDADMAC electrodes in an aqueous solution of 0.1 M Trizma buffer (pH 8.44). The thickness of the deposited films was approximately 200 µm.

Fig. S7 presents a schematic representation of the mediated electron transfer (MET) process between ferrocene species confined within the hybrid silica film and cytochrome c (Cyt c) in solution. The Fc@hybrid silica-modified electrode, prepared as described in Figure S5, was immersed in a 1 mg mL^{-1} Cyt c solution in PBS buffer. Cyclic voltammetry was then performed to assess the ability of the immobilized ferrocene to facilitate electron transfer to dissolved Cyt c.

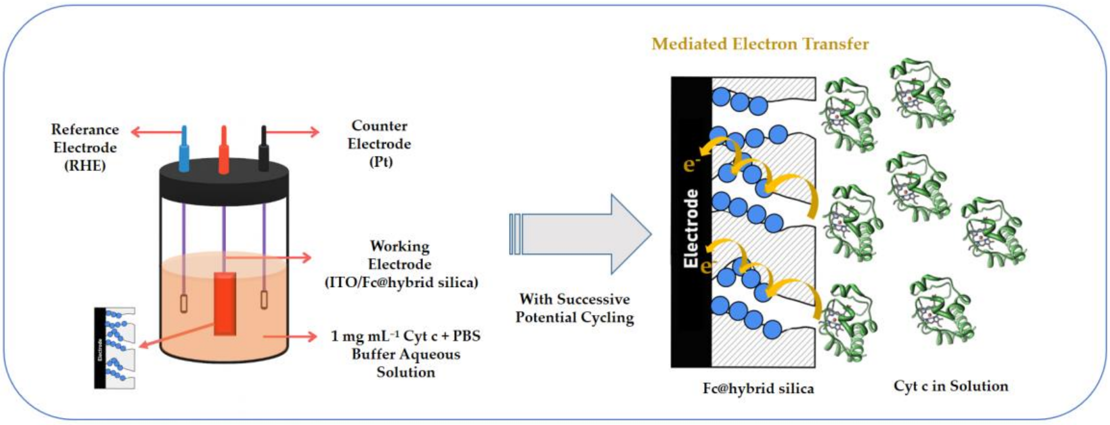

Figure S7. Schematic representation of the mediated electron transfer process between ferrocene species confined within the hybrid silica film and cytochrome c (Cyt c) in solution.

Fig. S8 shows the stabilized cyclic voltammograms of the silica-PSS-modified electrode recorded in PBS buffer (pH 7.4) and PBS containing 1 mg mL^{-1} cytochrome c. In both cases, no redox peaks are observed, indicating the absence of direct electron transfer between Cyt c and the electrode. Although electrostatic interactions may exist between the negatively charged silica-PSS film and the positively charged Cyt c (which has an isoelectric point around 10[38]) , the relatively small diameter of Cyt c (~3 nm) may limit its diffusion into the mesoporous silica network, further restricting its access to the electrode surface and contributing to the lack of electrochemical response.

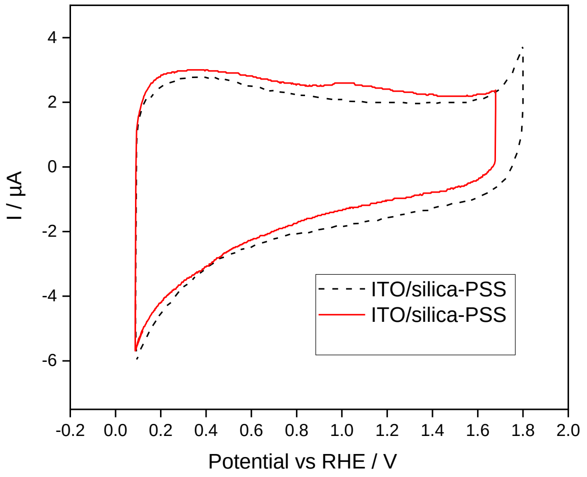

Figure S8. Stabilized cyclic voltammograms of the silica-PSS-modified electrode recorded in PBS buffer solution (pH 7.4, dashed line) and in PBS containing 1 mg mL^{-1} cytochrome c (solid line). Scan rate: 100 mV s^{-1}. The deposited film had an approximate thickness of 200 µm.
